# Statins can benefit patients with primary membranous nephropathy on venous thromboembolism

**DOI:** 10.1080/0886022X.2021.1879853

**Published:** 2021-02-15

**Authors:** Peimei Zou, Hang Li, Jianfang Cai, Zhenjie Chen, Chao Li, Xuewang Li

**Affiliations:** a Department of Nephrology, Peking Union Medical College Hospital, Chinese Academy of Medical Sciences and Peking Union Medical College, Beijing, China; b Department of Nephrology, Dongzhimen Hospital, Beijing University of Chinese Medicine, Beijing, China

**Keywords:** Primary membranous nephropathy, statin, venous thromboembolism

## Abstract

**Objective:**

The aim of this study was to investigate the role of prophylactic use of statin in venous thromboembolism (VTE) in patients with primary membranous nephropathy (PMN).

**Methods:**

A total of 734 patients with PMN were consecutively enrolled in this retrospective study. 564 patients had received statins prescription, while 170 patients did not. Kaplan–Meier methods were used for cumulative incidence plots of thromboembolic events and Cox proportional hazards regression models were used to assess risk factors. Finally, the effects of different potency of statins were evaluated.

**Results:**

In the cohort, 37 patients (5.0%) experienced VTE. In a univariate Cox proportional hazard model, the hazard ratio (HR) for VTE in statin users versus statin non-users was 0.5 (95% CI 0.3–0.8, *p* = .03). Multivariable model proportional-hazards analysis corrected for co-medications and risk factors revealed that adjusted HR was 0.4 (95% CI 0.1–0.7, *p* = .03). According to the type and dose, statin users were assigned into 3 groups: high-intensity group (*n* = 278), moderate-intensity group (*n* = 186), and low-intensity group (*n* = 49). In comparison, incidences of VTEs in the three groups were similar (2.9% vs 4.8% vs 2.0%, *p* = .45).

**Conclusions:**

The prophylactic use of statins could effectively decrease the occurrence of VTE in patients with PMN, and the benefits have no difference in different potency of statins.

## Introduction

Venous thromboembolism (VTE) is a common complication of nephrotic syndrome (NS), including deep vein thrombosis (DVT), renal vein thrombosis (RVT), and pulmonary embolism (PE) [1,2]. Primary membranous nephropathy (PMN), the most common cause of nephrotic syndrome in adult, thought to be at highest risk of thromboembolic events [3–5]. The mechanism underlying is incompletely understood. It is generally recognized that venous thromboembolism is associated with a hypercoagulable state attributed to the urinary loss of pro- and anticoagulant proteins such as antithrombin III, and increased production of fibrinogen in the liver under the conditions of proteinuria or hypoalbuminemia. Increased plate activation and aggregation involved in it as well. Hyperlipidemia, which also has been related to increased endothelial dysfunction, aggravates the hypercoagulable state [1,3,6–8]. VTE is a serious complication and increases the mortality of patients with PMN [9]. However, the most effective method of VTE prophylaxis is still unclear [10,11].

Statins, the HMG-CoA reductase inhibitors, are also effective in treating the hyperlipidemia of NS [12,13]. Several large-scale clinical trials in non-renal populations have demonstrated that statins can prevent atherosclerosis and decrease risks of cardiovascular diseases, even in individuals with normal blood lipid levels [14–17]. These means statins play the role of preventing atherosclerosis not only by their lipid-lowing properties, but also involve lipid-independent pleiotropic effects. It was supposed that statins may exert anti-inflammatory effects, enhancing vascular endothelial function, modulating the coagulation cascade and exhibiting anti-thrombotic properties, and reducing the formation of thrombin due to inhibition of platelet-derived protease-activated receptor 1 (PAR-1) and tissue factor up-regulation [18–20]. Many trails in healthy population [21–23] and studies involve cancer patients [24] found that the use of statins was associated with a reduction in the occurrence of VTE. Mohammad Resh *et al* demonstrated that statin use in patients with NS is associated with a lower risk of VTE [6]. In patients with PMN, study about the effect of statins to VTE is still lacked. Thus, we designed this study to investigate the role of prophylactic use of statin in VTE in patients with PMN.

## Materials and methods

This study was approved by the ethics committee of the Peking Union Medical College Hospital (NO. S-K120). The materials and methods were very similar to one of our published study conducted by the same team [25]. Patients pathologically diagnosed as PMN by renal biopsy from January 2004 to June 2016 at Peking Union Medical College Hospital were retrospectively enrolled. The exclusion criteria were also the same [25]. We excluded patients with malignant tumors, autoimmune diseases, serious mental diseases and hematological diseases. Patients with comorbidity of other pathological types of glomerular diseases and patients who presented with venous thrombotic events at the time of diagnosis of PMN were also excluded. Nephrotic syndrome (NS) was identified as proteinuria ≥3.5 g/24h and hypoalbuminemia (≤30 g/L) [26]. Clinical characteristics at the time of biopsy were collected, including gender, age, duration of the disease, history of smoking, diabetes and hypertension, proteinuria, serum albumin, serum creatinine, total cholesterol, triglycerides, and low-density lipoprotein-cholesterol (LDL-C) [25]. The estimated glomerular filtration rate (eGFR) was calculated using the Chronic Kidney Disease Epidemiology Collaboration (CKD-EPI) formula[27]. In addition, we also collected treatments for PMN, including angiotensin converting enzyme inhibitors (ACEIs) or angiotensin receptor blockers (ARBs), glucocorticoids, aspirin and the anticoagulant therapy. Statin regimens of patients were recorded. Medical records were reviewed to collect VTEs during statins treatment. Definitions of the potency of statins were: (1) high-intensity: rosuvastatin ≥10mg, atorvastatin ≥20mg, simvastatin ≥40mg; (2) moderate-intensity: rosuvastatin 5 mg, atorvastatin 10 mg, simvastatin 20 mg, pravastatin 40 mg or fluvastatin 80 mg; (3) low-intensity: simvastatin 10 mg, pravastatin 20 mg or fluvastatin 40 mg [28]. The diagnoses of DVT and RVT were confirmed by compression sonography and color-Doppler ultrasound. The diagnosis of PE was performed by CT pulmonary angiography [25].

We used SPSS (version 22, IBM, Armonk, NY, U.S.) to do the statistical analysis. Continuous variables were presented as mean ± SD or median with interquartile ranges (IQR), and the differences were evaluated by Student t-test, Mann–Whitney U test, Kruskal–Wallis test, or univariate ANOVA. Categorical variables were presented with percentages, and the differences were compared using chi-square test or Fisher’s test. Kaplan–Meier methods were used for cumulative incidence plots. To assess risk factors for VTE, we used Cox proportional hazards regression models. Baseline variables were incorporated into the models. A two-tailed *p* value of less than .05 was considered statistically significant.

## Results

### Baseline characteristics of study subjects

From January 2004 to June 2016, a total of 734 patients (58% were male) were enrolled in the study. The mean age was 47.4 ± 14.8 years. Median observation period was 39.6 (25.0, 62.1) months. 37 patients (5.0%) experienced VTE during the observation period. 564 patients received statins therapy for at least 1 month (statin + group), while 170 patients did not use statins (statin- group). Table 1 showed the baseline characteristics of both 2 groups. In statin + group, patients were older (48.4 ± 14.3 vs 44.0 ± 16.1 years, *p* = .001), serum albumin level was lower (26.9 ± 6.5 vs 29.0 ± 7.0 g/L, *p* = .001), eGFR level was lower (95.87 ± 21.50 vs 101.41 ± 23.32 mL/min/1.73m^2^, *p* = .004), and more patients had hypertension history (53% vs 42%, *p* = .01).

**Table 1.** Baseline characteristics of patients in each group.

	statin+ (*n* = 564)	statin− (*n* = 170)	*p* Value
Male, n (%)	322 (57)	104 (61)	.34
Age, y	48.4 ± 14.3	44.0 ± 16.1	.001
History of			
Smoking, *n* (%)	171 (30)	45 (26)	.33
Diabetes, *n* (%)	72 (13)	17 (10)	.33
Hypertention, *n* (%)	300 (53)	72 (42)	.01
Observation time, m	40.8 (27.0, 62.8)	32.9 (20.5, 57.3)	.003
Proteinuria, g/d	5.72 (3.49, 8.90)	4.23 (2.49, 7.25)	<.001
Serum albumin, g/l	26.9 ± 6.5	29.0 ± 7.0	.001
eGFR, ml/min/1.73m^2^	95.87 ± 21.50	101.41 ± 23.32	.004
Total cholesterol, mmol/l	7.79 ± 2.91	6.62 ± 2.51	<.001
Triglycerides, mmol/l	2.42 (1.74, 3.38)	2.30 (1.61, 3.63)	.64
LDL-C, mmol/l	5.15 ± 2.08	4.44 ± 1.86	.01

eGFR: estimated glomerular filtration rate; LDL-C: low density lipoprotein cholesterol.

### Risk of VTE

**Figure 1.**
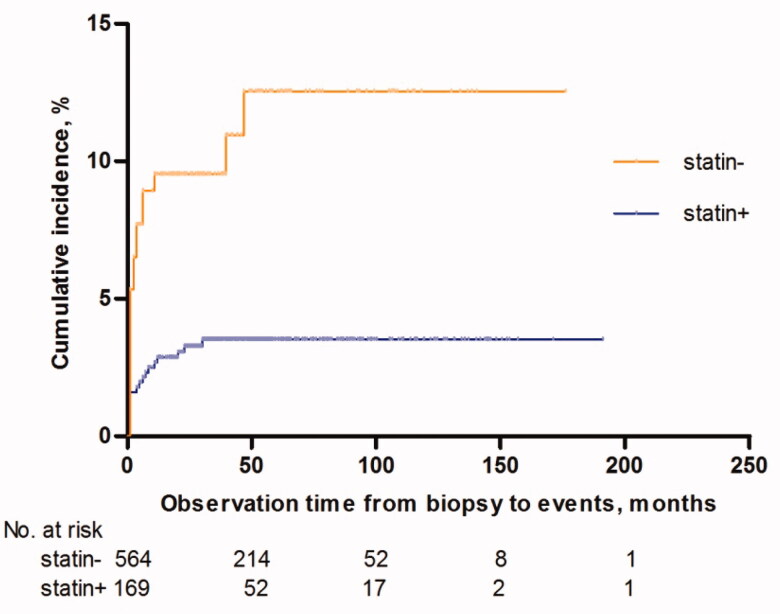
Kaplan–Meier estimates of the probability of VTEs-cumulative incidences.

In statin + group and statin- group, 19 patients (3.4%) and 18 patients (10.6%) (*p* < .001) experienced VTE during the observation period, respectively. The cumulative incidence rates of VTEs in the 2 groups were exhibited in Figure 1.

Table 2 showed the distribution of drugs use across the two groups. Statin users used more often glucocorticoids (*p* < .001), ACEI/ARB (*p* < .001) and aspirin (*p* < .001). No significant difference was found for LMWH/warfarin use between the two groups.

**Table 2.** Drug exposure compared between statin + group and statin − groups.

	statin+ (*n* = 564)	statin− (*n* = 170)	*p* Value
GCC	495 (88)	119 (70)	<.001
ACEI/ARB	485 (86)	119 (70)	<.001
LMWH/warfarin	59 (10)	11 (6)	.13
Aspirin	101 (18)	13 (8)	.001

GCC: glucocorticoids; ACEI: angiotensin converting enzyme inhibitors; ARB: angiotensin receptor blockers; LMWH: low molecular weight heparin.

In a univariate Cox proportional hazard model, statin use was associated with lower risk of VTE (HR 0.5 (95% CI 0.3–0.8), *p* = .03) (Table 3). Multivariate analysis corrected for co-medications (including glucocorticoids, ACEI/ARB and aspirin) and the serum albumin level did not change the outcome, adjusted hazard ratio was 0.4 (95% CI 0.1–0.7, *p* = .03).

**Table 3.** Univariate and multivariable model proportional-hazards analysis on risk factors of VTE.

	Univariate analysis		Multivariable analysis^a^	
	HR (95% CI)	*p* Value	HR (95% CI)	*p* Value
Statin	0.5 (0.3–0.8)	.03	0.4 (0.1–0.7)	.03
GCC	1.0 (0.4–2.4)	.96		
ACEI/ARB	0.3 (0.2–0.6)	<.001		
LMWH/warfarin	0.6 (0.2–1.9)	.39		
Aspirin	0.5 (0.1–1.5)	.21		

HR: hazard ratio; GCC: glucocorticoids; ACEI: angiotensin converting enzyme inhibitors; ARB: angiotensin receptor blockers; LMWH: low molecular weight heparin.

**^a^**Adjusted for co-medications and the serum albumin level.

### Effects of different potency of statins

Among 564 statin users, details about the statin types of 51 patients were unavailable. In the 51 patients, one patient occurred VTE. According to the type and dose, the remaining 513 statin users were divided into 3 groups: high-intensity group (*n* = 278), moderate-intensity group (*n* = 186) and low-intensity group (*n* = 49). As showed in Table 4, baseline characteristics had no differences in three groups. In comparison, incidences of VTEs in the three groups were similar (2.9% vs 4.8% vs 2.0%, *p* = .45).

**Table 4.** Baseline characteristics and effects on VTE in different potency of statins.

	High-intensity (*n* = 278)	Moderate-intensity (*n* = 186)	Low-intensity (*n* = 49)	*p* Value
**Baseline**				
Male, n (%)	164 (59)	89 (48)	34 (69)	.01
Age, y	48.0 ± 14.1	49.5 ± 14.8	49.1 ± 14.0	.50
History of				
Smoking, n (%)	90 (32)	49 (26)	18 (37)	.24
Diabetes, n (%)	41 (15)	27 (15)	4 (8)	.46
Hypertention, n (%)	151 (54)	99 (53)	26 (53)	.97
Proteinuria, g/d	5.66 (3.60, 8.73)	5.60 (3.01, 8.96)	6.24 (4.08, 12.68)	.17
Serum albumin, g/l	26.8 ± 6.1	27.7 ± 7.1	25.3 ± 5.3	.07
eGFR, ml/min/1.73m^2^	96.69 ± 22.10	93.71 ± 21.19	96.40 ± 19.85	.33
Total cholesterol, mmol/l	7.75 ± 3.05	7.86 ± 2.86	7.75 ± 2.27	.93
Triglycerides, mmol/l	2.46 (1.78, 3.48)	2.35 (1.70, 3.28)	2.36 (1.78, 3.32)	.68
LDL-C, mmol/l	5.13 ± 2.18	5.22 ± 2.08	5.16 ± 1.67	.94
**VTE**, n (%)	8 (2.9)	9 (4.8)	1 (2.0)	.45

eGFR: estimated glomerular filtration rate; LDL-C: low density lipoprotein cholesterol; VTE: venous thromboembolism.

## Discussion

To our knowledge, this is the first study that investigates the role of statin in VTE in PMN, and further evaluated the effects of different potency of statins. Our study results suggest that statin use is associated with a lower risk of VTE in patients with PMN, and the benefits have no difference in different potency of statins. It means such low-intensity statin that can exhibit the protective effect of VTEs in PMN.

Our previous study with the same cohort indicated that patients with PMN have increased incidences of arterial thromboembolic events (ATEs) and VTEs, with most of events occurred within the first 6 months of the disease. In 60 VTEs, the deep vein thrombosis (DVT), renal vein thrombosis (RVT) and pulmonary embolism (PE) accounted for 60%, 13% and 27% respectively. Massive proteinuria and hypoalbuminemia were associated with VTEs, and hypoalbuminemia was the dominant independent risk factor (*p* < .03) [29]. Thus, in the multivariate analysis of this study, we corrected for co-medications and risk factors for VTEs we demonstrated above. Adjustments did not change the outcome.

Statins are known to have pleiotropic effects on coagulation and inflammation, including improving endothelial function, decreasing oxidative stress and inflammation, enhancing stability of atherosclerotic plaques, decreasing platelet activation, inhibiting thrombosis, and inhibition of smooth muscle proliferation [19,30]. The Heart and Estrogen/progestin Replacement Study (HERS) in 2002 initially discussed the effect of statins on primary prevention of VTE [31]. It was a randomized clinical trial to evaluate the effects of estrogen and progesterone supplementation on cardiovascular events in 2763 postmenopausal women with coronary heart disease. In a nonrandomized comparison of statin versus non-statin users, an approximately 50% risk reduction in VTE was reported. The Justification for the Use of Statin in Prevention: an Intervention Trial Evaluating Rosuvastatin (JUPITER) trial, which is the first randomized controlled trial (RCT), results of its 17802 participants showed that rosuvastatin significantly reduced the occurrence of VTEs [17]. However, over the past decade, several publications on the topic had inconsistent results [32,33]. In 2012, Rahimi *et al* conducted a meta-analysis included 29 RCTs, found no significant reduction in VTEs with statin treatment [34]. In 2017, Kunutsor *et al* did a systematic review and meta-analysis of 13 observational cohort studies involved 3148259 participants and 23 RCTs involved 118464 participants, suggested a beneficial effect of statin use on venous thromboembolism, and therapy with rosuvastatin significantly reduced venous thromboembolism compared with other statins [35].

It was demonstrated that patients with PMN were at high risk of VTEs, and massive proteinuria and hypoalbuminemia were the risk factors [5,29]. In our cohort, statin users were older aged and tended to be severe nephrosis, which presented with severe proteinuria, severe hypoalbuminemia, and lower eGFR. It indicated that risks of VTE in statin + group patients were higher. Results of our study revealed statin use in patients with PMN was associated with low risk of VTE, corroborated the beneficial effect of statin use on venous thromboembolism. Not very consistent with the study of Kunutsor et al. [35], we found the benefits have no difference in different potency of statins. Therefore, statins may be considered a good choice in some patient populations who are not suitable candidates for anticoagulant therapy to minimize the associated risk of bleeding.

There are also several limitations in this study. Firstly, it is a retrospective observation study, baseline laboratory data were not available in all patients. Secondly, asymptomatic VTEs may have been missed because participants were not routinely screened, which can lead to the underestimate of the thromboembolism incidence. Finally, records of side effects associated with statins were lacked. In conclusion, our retrospective data suggest that prophylactic use of statins can effectively decreased the occurrence of VTE in patients with PMN, and the benefits have no difference in different potency of statins. Further research is required to validate the benefits of statin therapy on venous thromboembolism.
